# NFE2L3 Inhibition Induces Cell Cycle Arrest at the G0/G1 Phase in Colorectal Cancer Cells through Downregulating CCND1 and pRb1-ser807/811

**DOI:** 10.1155/2019/2829798

**Published:** 2019-05-05

**Authors:** Lihua Zhang, Dong-Li Hu, Baiyou Tang, Yu Cheng, Chuan Jiao, Lin Cheng, Zhi-Rong Tan, Hong-Hao Zhou

**Affiliations:** ^1^Department of Clinical Pharmacology, Xiangya Hospital, Central South University, Changsha 410008, China; ^2^Institute of Clinical Pharmacology, Central South University, Hunan Key Laboratory of Pharmacogenetics, Changsha 410078, China; ^3^Center for Medical Genetics, Central South University, Changsha, Hunan 410012, China; ^4^State Key Laboratory of Ophthalmology, Zhongshan Ophthalmic Center, Sun Yat-sen University, 54 South Xianlie Road, Guangzhou, Guangdong, China

## Abstract

The molecular mechanism for colorectal cancer to develop remains unelucidated. To find biomarkers related to colorectal cancer development, we analyzed the gene expression profile of 380 colorectal cancer patients and 51 healthy controls by R software. Finally, 1579 upregulated differential expression genes (DEGs) and 3218 downregulated DEGs were identified. Then, the top 20 upregulated DEGs were compared with 181 upregulated DEGs that we reported previously, and 11 overlapped DEGs were found. *NFE2L3* (nuclear factor, erythroid 2-like 3) was among those overlapped DEGs and was rarely reported in colorectal cancer. Real-time polymerase chain reaction (PCR) results showed that higher *NFE2L3* expression levels were identified in paired tumor samples than in paratumor samples (48 paired samples). Flow cytometry analysis revealed that the cell cycle was arrested at the G0/G1 phase after inhibition of *NFE2L3* in both HCT116 and SW480 cell lines. Western blot detection showed that CCND1 and phosphorylated Rb transcriptional corepressor 1 at ser-807/811 (pRb1-ser807/811) expression levels were downregulated when NFE2L3 was inhibited in those two cell lines. A significant positive correlation was observed between *NFE2L3* and *CCND1* expression levels in colorectal tissue samples. These evidences indicate that downregulation of *NFE2L3* induces cell cycle arrest at the G0/G1 phase through downregulation of CCND1 and pRb1-ser807/811.

## 1. Introduction

Colorectal cancer is a fatal threat to human health worldwide. Colorectal cancer ranks third place in all cases of malignant tumor diagnosis in the US [1]. Although a few risk factors can result in this disease, the underlying mechanism is not clearly elucidated yet.

Nowadays, some common genes related to colorectal cancer development had been reported. Somatic mutations were common ones in colorectal cancer, in which *TP53* [2, 3], *APC* [4, 5], *KRAS* [6], and *BRAF* [7, 8] mutations are the most frequently reported. Epigenetic changes are another common pathogenesis that relates to tumorigenesis and progression. Abnormal methylation of septin 9 (*SEPT9*) is one of the most frequently reported events [9, 10], and the *SEPT9* methylation test has already been approved by the Food and Drug Administration in early screening for colorectal carcinoma [11]. Gene expression aberrance is another important predictor for colorectal carcinogenesis.

In recent years, numbers of tumor-related datasets have been shared on databases, such as Gene Expression Omnibus (GEO, https://www.ncbi.nlm.nih.gov/geo/) and The Cancer Genome Atlas (TCGA, https://cancergenome.nih.gov/). Meanwhile, several convenient tools have been developed to do dataset analysis, in which R was one of the most commonly used ones.

To search biomarkers in the gene expression level, we downloaded an expression dataset named COADREAD (Illumina HiSeq) from TCGA database. After analysis with R software, 1579 significantly upregulated DEGs and 3218 downregulated DEGs were identified. Then, the top 20 upregulated DEGs were merged and compared with our previously reported 181 common upregulated DEGs which were derived from 59 paired tumor and nontumor samples [12]. 11 common DEGs were eventually found, among which a transcription factor gene named *NFE2L3* attracted our interest.

The human gene *NFE2L3* was first identified in 1999 and has been reported to be associated with oxidative stress and the Wnt signaling pathway [13–15]. The *NFE2L3* messenger RNA (mRNA) is also identified to be upregulated in thyroid cancer and preinvasive testicular carcinoma samples [16, 17]. In thyroid cancer cells, NFE2L3 overexpression promotes cell proliferation and invasion [17]. In colorectal cancer, NFE2L3 is reported to promote cell proliferation [18]. Nevertheless, there is no report on the mechanism of *NFE2L3* in cell cycle regulation. In this study, we verified that *NFE2L3* mRNA expression was upregulated in colorectal cancer samples when compared to adjacent samples. Then, the cell cycle was arrested at the G0/G1 phase after knockdown of *NFE2L3* in HCT116 and SW480 cell lines, as reflected by flow cytometry. This probably resulted from the downregulation of CCND1 and pRb1-ser807/811, which are the two key cell cycle regulatory factors in the G1 phase.

## 2. Material and Methods

### 2.1. Microarray Data

To search for biomarkers related to colorectal cancer development at the expression level, we searched the UCSC Cancer Browser (https://genome-cancer.ucsc.edu/proj/site/hgHeatmap/) and downloaded a TCGA colorectal cancer dataset named COADREAD gene expression (Illumina HiSeq) with 434 samples. Four sample types were included in the dataset, including metastatic tumor (*n* = 1), recurrent tumor (*n* = 2), primary tumor (*n* = 380), and normal sample (*n* = 51).

### 2.2. Identification of DEGs

Primary tumor and normal healthy control samples were selected for analysis. At first, clustering analysis was performed by R software in the primary tumor and normal samples. Then, the probability of genes being differentially expressed between primary tumor and normal samples was detected by *limma* function in the R program. The DEGs were selected with the criteria of corrected FDR < 0.05 and ∣fold change∣ ≥ 2.0. Finally, the volcano plot was depicted by R [19, 20].

### 2.3. Tissue Samples

Tumor tissue samples and individual-matched adjacent mucosa samples were obtained from 48 patients with colorectal cancer who underwent resection surgery at Xiangya Hospital between 2014 and 2017. The adjacent mucosa samples were acquired 2-5 cm away from the tumor. Tissue samples were stored immediately in liquid nitrogen after surgery. Our study was approved by the Institutional Review Board of the Department of Clinical Pharmacology, Xiangya Hospital, Central South University.

### 2.4. Cell Culture and Transfection

HCT116 and SW480 cells were cultured in RPMI 1640 medium supplemented with 10% fetal bovine serum (FBS) and incubated at 37°C in a humidified chamber with 5% CO_2_.

The transfection of short interfering RNA (siRNA) was carried out using RNAiMAX (Invitrogen). The final concentrations of the siRNA were 100 nM. After transfection for 48 hours, the cells were utilized for experiments. The sequences of the siRNA were as follows: for the negative control (NC), 5′- UUCUCCGAACGUGUCACGUTT-3′; for siRNA-1 (si-1), 5′-GCACGAAGCUGUGGAUCAUTT-3′; and for siRNA-2 (si-2), 5′-GACUUAUUCCAGUUGCUUUTT-3′.

### 2.5. RNA Isolation, Reverse Transcription, and Real-Time PCR

Tissue specimen and cell total RNAs were extracted with the RNAiso Plus reagent (Takara). Then, reverse transcription was done with the PrimeScript 1st Strand cDNA Synthesis Kit (Takara). The real-time PCR assay was performed on the LightCycler 480II (Roche) platform with SYBR Premix DimerEraser kit (Takara). PPIA and B2M genes were set as the internal controls in tissue specimens. Actin served as the internal control in cell samples. All primer sequences are presented in Table S1.

### 2.6. Cell Cycle Analysis by Flow Cytometry

SW480 and HCT116 cells were serum starved for 12 hours before the transfection. After transfection for 48 hours, the transfected cells were trypsinized, washed, and resuspended in precooled 70% ethanol solution. The cells were resuspended using PBS and incubated with RNase and propidium iodide (PI) for 30 minutes at room temperature before testing. The cells were detected with an FC500 flow cytometer (Beckman). This experiment was repeated in triplicate, and the data were analyzed with ModFit software (Verity Software House).

### 2.7. Western Blot

After transfection for 48 h, total proteins were extracted using radioimmunoprecipitation assay buffer with the protease inhibitor. Proteins were separated by 10% sodium dodecyl sulfate-polyacrylamide gel electrophoresis and then transferred onto polyvinylidene fluoride membranes. The primary antibodies were incubated at 4°C. After incubation with secondary antibodies at room temperature for 1 h, the proteins were visualized and quantified by Quantity One software (Bio-Rad) [21]. Antibodies against CCND1 and pRb1-Ser807/811 were obtained from Cell Signaling Technology. An NFE2L3 antibody was obtained from Boster Biological Technology. An actin protein antibody was purchased from Sigma. This experiment was repeated in triplicate.

### 2.8. Statistical Analysis

Statistical analyses were performed with GraphPad Prism 6.0 software (GraphPad Software Inc.) [22]. The real-time PCR data of tissue samples and cells were analyzed with 2^-ΔCt^ and 2^-ΔΔCt^, respectively. Student's *t*-test was used to evaluate the significance of the groups, and *p* < 0.05 was considered statistically significant.

## 3. Results

### 3.1. Identification of DEGs and NFE2L3 Expression Levels in Colorectal Cancer Cells

**Figure 1:**
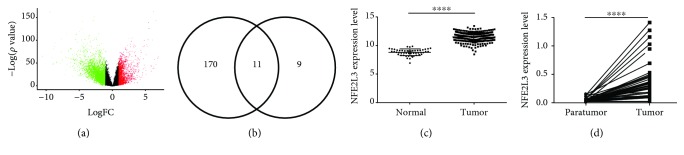
Screening and validation of differential expression genes. (a) Volcano plot of differential expression genes in TCGA COADREAD gene expression dataset. *x*-axis: log2 fold change; *y*-axis: -log10 (FDR *p* value) for each probe; vertical dotted lines: fold change ≥ 2 or ≤2. It showed that 1579 significantly upregulated genes (red color) and 3220 significantly downregulated genes (green color) were selected in this dataset. (b) 11 merged genes were found with the intersection of the top 20 upregulated genes from the COADREAD dataset and 181 upregulated genes from our previous research. (c) *NFE2L3* expression levels in 51 normal and 370 tumor samples. *NFE2L3* showed significantly higher expression levels in tumor samples than in normal samples (*p* < 0.0001). (d) Validation of *NFE2L3* expression levels with 48 paired tumor and paratumor samples. The *NFE2L3* mRNA expression level was significantly upregulated in tumor samples (*p* < 0.0001).

Sample clustering analysis showed that the primary tumor samples and normal samples were well separated (Figure S1). DEG analysis detected 1579 significantly upregulated DEGs and 3218 drastically downregulated DEGs (Figure 1(a)). All of the DEGs are presented in Table S2. To search for a biomarker related to tumorigenesis, we got 11 overlapped DEGs (*CDH3*, *KRT80*, *ETV4*, *ESM1*, *FOXQ1*, *CLDN1*, *NFE2L3*, *LRP8*, *TEAD4*, *TRIB3*, and *WNT2*) from 181 upregulated DEGs related to colorectal cancer development in our previous report (Figure 1(b)) and the top 20 upregulated DEGs in this analysis. After screening, the transcription factor *NFE2L3* attracted our interest. In the COADREAD expression dataset, *NFE2L3* was markedly upregulated in colorectal cancer samples compared with normal samples (Figure 1(c), *p* < 0.0001), validated by our real-time PCR assay in 48 paired paratumor and tumor samples (Figure 1(d), *p* < 0.0001).

### 3.2. *NFE2L3* Knockdown Arrests Cell Cycle Progression in HCT116 and SW480 Cell Lines

**Figure 2:**
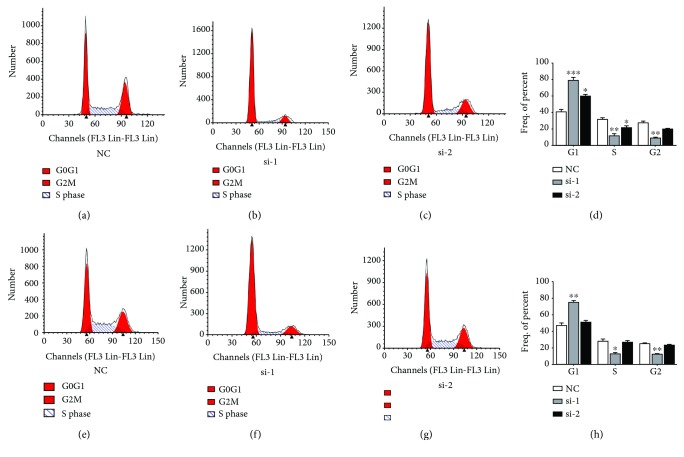
Changes in cell cycle distribution in HCT116 and SW480 cells after transfection with siRNA for 48 hours. (a–d) Cell cycle distribution in the HCT116 cell line. In HCT116 cells, the cell percentage in NC, si-1, and si-2 groups was 41.07 ± 5.18, 79.14 ± 5.75, and 59.53 ± 4.59, respectively, at the G0/G1 phase; 31.68 ± 2.98, 11.59 ± 4.77, and 21.08 ± 5.37, respectively, at the S phase; and 27.25 ± 2.79, 9.27 ± 1.52, and 19.4 ± 2.06 at the G2 phase. The cell percentage of the si-1 and si-2 groups at the G0/G1 phase was reduced compared to that of the NC group (*p* = 0.0003, *p* = 0.0216). The cell percentage at the S phase was decreased in the si-1 and si-2 groups compared to the NC group (*p* = 0.0044, *p* = 0.0180). The cell percentage at the G2/M phase was reduced in the si-1 group compared to the NC group (*p* = 0.0026). (e–h) Cell cycle distribution in the SW480 cell line. In SW480 cells, the cell percentage in NC, si-1, and si-2 groups was 47.18 ± 4.56, 74.77 ± 3.31, and 50.39 ± 3.65, respectively, at the G0/G1 phase; 27.70 ± 5.65, 12.80 ± 2.64, and 26.56 ± 3.98, respectively, at the S phase; and 25.12 ± 1.09, 12.43 ± 0.79, and 23.05 ± 1.38, respectively, at the G2 phase. The cell percentage at the G0/G1 phase was reduced in the si-1 group compared to the NC group (*p* = 0.0012). The cell percentage at the S phase was decreased in the si-1 group compared to the NC group (*p* = 0.0136). The cell percentage at the G2/M phase was reduced in the si-1 group compared to the NC group (*p* = 0.0064).

We use PI staining and flow cytometry to detect the effect of *NFE2L3* knockdown on the cell cycle in HCT116 and SW480 cells after 48 h transfection. In HCT116 cells (Figures 2(a)–2(d)), the cell percentages of the si-1 and si-2 groups were raised significantly compared with that of the normal control (NC) group at the G0/G1 phase (*p* = 0.0003, *p* = 0.0216). The S phase cell percentages of the si-1 and si-2 groups were reduced significantly in comparison to that of the NC group (*p* = 0.0044, *p* = 0.0180). The cell percentage of the NC group was considerably higher than that of the si-1 group at the G2/M phase (*p* = 0.0026). In SW480 cells (Figures 2(a)–2(d)), the cell percentage of the si-1 group was considerably higher than that of the NC group at the G0/G1 phase (*p* = 0.0012). At S and G2/M phases, the cell percentage of the si-1 group was reduced significantly compared with that of the NC group (*p* = 0.0136, *p* = 0.0064).

### 3.3. *NFE2L3* Knockdown Results in Repression of CCND1 and pRb1-ser807/811

To search for the mechanism for cell cycle changes, real-time PCR and western blot assays were conducted after transfection with NFE2L3 siRNA for 48 h.

**Figure 3:**
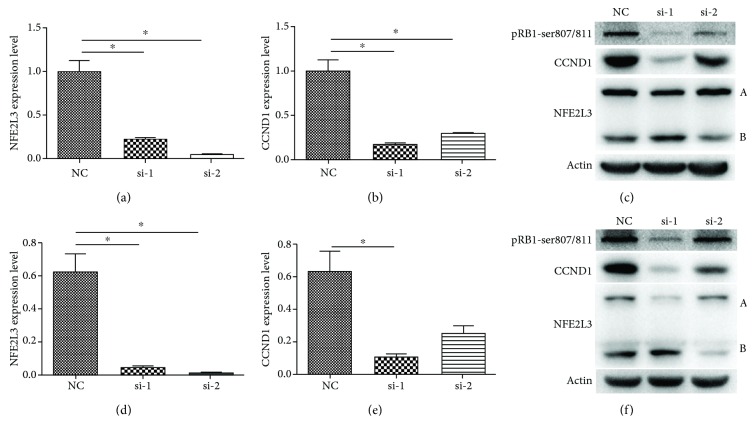
*CCND1* mRNA and protein expression levels in colorectal cancer cell lines after *NFE2L3* knockdown. (a) The mRNA expression levels of *NFE2L3* in HCT116 cells after *NFE2L3* knockdown. Inhibition of the *NFE2L3* mRNA level was displayed in the si-1 and si-2 groups (*p* = 0.0225, *p* = 0.0184). (b) *CCND1* mRNA expression levels in HCT116 cells after *NFE2L3* knockdown. Inhibition of *CCND1* mRNA expression was detected in the si-1 and si-2 groups (*p* = 0.0189, *p* = 0.0291). (c) Protein expression changes of CCND1 in HCT116 cells when inhibited by *NFE2L3* siRNA. Downregulation of CCND1 and pRb1-ser807/811 proteins was observed in the si-1 and si-2 groups. (d) The mRNA expression levels of *NFE2L3* in SW480 cells after *NFE2L3* knockdown. The *NFE2L3* mRNA expression level was significantly inhibited by si-1 and si-2 (*p* = 0.0258, *p* = 0.0232). (e) *CCND1* mRNA expression levels in SW480 cells after *NFE2L3* knockdown. The *CCND1* mRNA level was significantly downregulated in the si-1 group (*p* = 0.0374). (f) Protein expression changes of CCND1 in SW480 cells after transfection with *NFE2L3* siRNA. CCND1 and pRb1-ser807/811 protein expression levels were downregulated in the si-1 and si-2 groups.

In HCT116 cells, the *NFE2L3* mRNA level was dramatically downregulated in the si-1 and si-2 groups, as shown in Figure 3(a) (*p* = 0.0225, *p* = 0.0184). In Figure 3(b), real-time PCR results indicate that *CCND1*, which is a key cell cycle regulator, was downregulated in the si-1 and si-2 groups after *NFE2L3* knockdown (*p* = 0.0189, *p* = 0.0291). In Figure 3(c), two forms of *NFE2L3* proteins were detected and were named as A and B forms, respectively. Surprisingly, the expression level of the NFE2L3 A form was downregulated, but the B form was upregulated in the si-1 group. In the si-2 group, only the NFE2L3 B form was downregulated. No obvious change was observed in the A form. At the same time, pRb1-ser807/811 and CCND1 protein levels were downregulated after transfecting with NFE2L3 siRNA.

In SW480 cells, the mRNA level of *NFE2L3* and *CCND1* was also detected after transfection with *NFE2L3* siRNA. As shown in Figures 3(d)–3(e), *NFE2L3* mRNA was markedly downregulated in the si-1 and si-2 groups (*p* = 0.0258, *p* = 0.0232), and the *CCND1* mRNA level was significantly lower in the si-1 group (*p* = 0.0374). In Figure 3(f), the expression pattern of NFE2L3 proteins similar to that of HCT116 cells was found in the si-1 and si-2 groups. CCND1 and pRb1-ser807/811 proteins were also downregulated in two siNFE2L3 groups.

**Figure 4:**
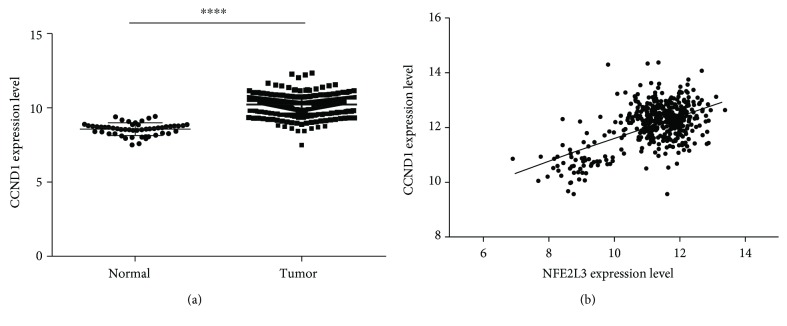
The expression correlation between *CCND1* and *NFE2L3* in colorectal tissue samples. (a) *CCND1* was upregulated in 380 tumor samples compared to 51 normal samples (*p* < 0.0001). (b) Expression level correlation analysis between *NFE2L3* and *CCND1* in 431 samples (normal and primary tumor samples) of TCGA COADREAD gene expression dataset. A significant positive correlation between *NFE2L3* and *CCND1* expression was found (*r* = 0.5698, *p* < 0.0001).

To find the relationship between *CCND1* and *NFE2L3* in colorectal tissue samples, the expression data of *CCND1* and *NFE2L3* were got from TCGA COADREAD gene expression dataset. As shown in Figures 4(a)–4(b), *CCND1* was significantly upregulated in tumor samples (*p* < 0.0001), and there was a significant positive expression correlation between *NFE2L3* and *CCND1* (*r* = 0.5698, *p* < 0.0001). This might suggest that the same case happened in colorectal cancer cells where CCND1 is regulated by NFE2L3 in colorectal tissue samples.

## 4. Discussion

*NFE2L3*, *NFE2*, *NFE2L1*, *NFE2L2*, *Bach1*, and *Bach2* are members of the cap‘n'collar (CNC) family in vertebrates [23, 24]. A CNC domain and a basic leucine zipper domain are common features of this protein family [25]. It is reported that knockout of the *Nfe2l3* gene in mice did not lead to fetal death during embryonic periods. There were no differences in phenotypes between *Nfe2l3*-null mice and wild-type littermates [23]. But *Nfe2l3*-null mice are more susceptible to lymphomagenesis than wild-type mice [26]. Interestingly, NFE2L3 is upregulated in human cancers, such as thyroid cancer and preinvasive testicular carcinoma [16, 17]. Only 68% Nfe2l3 amino acid homology is defined between mice and human, which indicates that NFE2L3 might not play the same role in these two species [25].

NFE2L3 protein is reported to perform the transcriptional function by binding with small Maf proteins to form heterodimers in the nucleus [25]. And more than one form of NFE2L3 protein has been found. Nouhi et al. report that three forms of NFE2L3 proteins are observed in HEK293T and JAR cells, among which the one with the largest molecular weight is proved to be glycosylated while the other two are not [27]. In our results of western blot, two forms of NFE2L3 proteins in HCT116 and SW480 cells were detected. These suggest that *NFE2L3* has more than one transcript. In our interference experiment for *NFE2L3*, two *NFE2L3* siRNAs showed a different effect on the two forms of NFE2L3 proteins. The si-1 mainly inhibited the expression of the A form, while the B form was upregulated. The si-2 only downregulated the expression level of the B form. We speculate that the si-1 and si-2 may not interfere with the same transcript. Further research is needed to explain this phenomenon.

Cyclin-dependent kinases (CDKs), cyclins, and Rb1 are important cell cycle regulators. In the mid to late G1 phase, CDKs bind to and activate cyclins, and then the complexes phosphorylate the tumor suppressor Rb1 [28]. CCND1 is the key cyclin that binds to CDK4/CDK6 in the G1 phase. Downregulation of CCND1 can induce cell cycle arrest in the G1 phase [28, 29]. The expression level of pRb1-ser807/811 is considered an indicator of cell cycle arrest in the G1 phase [30, 31]. In this research, CCND1 and pRb1-ser807/811 levels were downregulated after *NFE2L3* silencing in HCT116 and SW480 cells. Downregulation of CCND1 blocks the signals from CDK4/6, leading to pRb1-ser807/811 downregulation in both cell lines, which is responsible for cell cycle arrest at the G1 phase. Yet whether NFE2L3 directly regulates CCND1 needs further investigation.

In summary, we found that *NFE2L3* was upregulated in colorectal cancer. Knocking down *NFE2L3* could arrest the cell cycle at the G0/G1 phase in HCT116 and SW480 cells. We found that this inhibition is probably induced by CCND1 and pRb1-ser807/811 downregulation.

## Data Availability

All the data used to support the findings of this study are available from the first author upon request. And the email address of the first author is zhanglihua1107@csu.edu.cn.

## Supplementary Materials

Supplementary 1 Figure S1: clustering analysis of the 431 samples. In this figure, the sample similarity was displayed. All of the normal samples were clustered together. This reflects good sample consistency.

Supplementary 2 Table S1: primer sequences of the real-time PCR primers.

Supplementary 3 Table S2: 1579 upregulated genes and 3218 downregulated genes in colorectal cancer samples.
