# Supplementary figures and images for: NFE2L3 Inhibition Induces Cell Cycle Arrest at the G0/G1 Phase in Colorectal Cancer Cells through Downregulating CCND1 and pRb1-ser807/811
Source: Dis Markers. 2019 May 5;2019:2829798. doi: 10.1155/2019/2829798 (PMC6525936; doi:10.1155/2019/2829798)

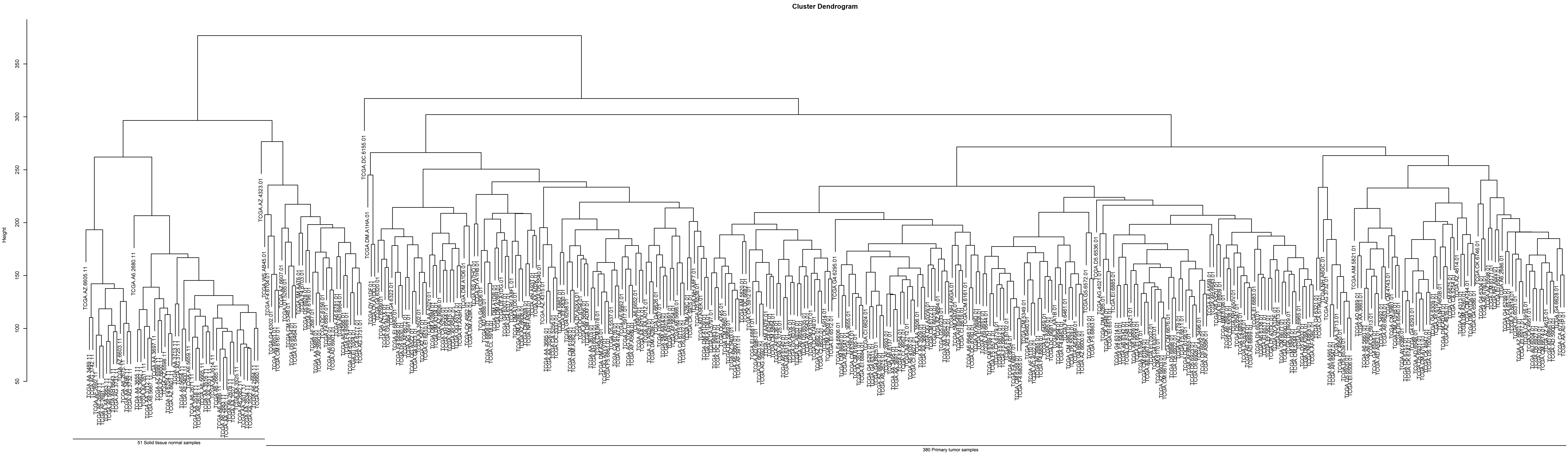

Supplement: Supplementary 1 — Figure S1: clustering analysis of the 431 samples. In this figure, the sample similarity was displayed. All of the normal samples were clustered together. This reflects good sample consistency. [file 2829798.f1.jpg]
