# Supplementary material for: NFE2L3 Inhibition Induces Cell Cycle Arrest at the G0/G1 Phase in Colorectal Cancer Cells through Downregulating CCND1 and pRb1-ser807/811
Source: Dis Markers. 2019 May 5;2019:2829798. doi: 10.1155/2019/2829798 (PMC6525936; doi:10.1155/2019/2829798)
Supplement: Supplementary 2 — Table S1: primer sequences of the real-time PCR primers. [file 2829798.f2.docx]

Table. 1. Primer sequences for real-time PCR.

| Gene name | Primer sequence |
| --- | --- |
| PPIA | F: 5^，^-GTGGTATAAAAGGGGCGGGAG- 3^，^ |
|  | R: 5^，^-GTGGGGTTGACCATGGCTAATAGTA- 3^，^ |
| B2M | F: 5^，^-ATGCCTGCCGTGTGAACCATGTGA- 3^，^ |
|  | R: 5^，^-TCCAAATGCGGCATCTTCAAACCTC- 3^，^ |
| actin | F: 5^，^- TGACTGACTACCTCATGAAGAT - 3^，^ |
|  | R: 5^，^- CATGATGGAGTTGAAGGTAGTT - 3^，^ |
| NFE2L3 | F: 5^，^- TGACTGGGAGGCAGAAAAGAC - 3^，^ |
|  | R: 5^，^- AATGAGATGCCCTCCAGTGA - 3^，^ |
